# Nutritional Basis for Colonization Resistance by Human Commensal *Escherichia coli* Strains HS and Nissle 1917 against *E. coli* O157:H7 in the Mouse Intestine

**DOI:** 10.1371/journal.pone.0053957

**Published:** 2013-01-17

**Authors:** Rosalie Maltby, Mary P. Leatham-Jensen, Terri Gibson, Paul S. Cohen, Tyrrell Conway

**Affiliations:** 1 Department of Microbiology and Plant Biology, University of Oklahoma, Norman, Oklahoma, United States of America; 2 Department of Cell and Molecular Biology, University of Rhode Island, Kingston, Rhode Island, United States of America; U. S. Salinity Lab, United States of America

## Abstract

*Escherichia coli* is a single species consisting of many biotypes, some of which are commensal colonizers of mammals and others that cause disease. Humans are colonized on average with five commensal biotypes, and it is widely thought that the commensals serve as a barrier to infection by pathogens. Previous studies showed that a combination of three pre-colonized commensal *E. coli* strains prevents colonization of *E. coli* O157:H7 in a mouse model (Leatham, et al., 2010, Infect Immun 77: 2876–7886). The commensal biotypes included *E. coli* HS, which is known to successfully colonize humans at high doses with no adverse effects, and *E. coli* Nissle 1917, a human commensal strain that is used in Europe as a preventative of traveler's diarrhea. We hypothesized that commensal biotypes could exert colonization resistance by consuming nutrients needed by *E. coli* O157:H7 to colonize, thus preventing this first step in infection. Here we report that to colonize streptomycin-treated mice *E. coli* HS consumes six of the twelve sugars tested and *E. coli* Nissle 1917 uses a complementary yet divergent set of seven sugars to colonize, thus establishing a nutritional basis for the ability of *E. coli* HS and Nissle 1917 to occupy distinct niches in the mouse intestine. Together these two commensals use the five sugars previously determined to be most important for colonization of *E. coli* EDL933, an O157:H7 strain. As predicted, the two commensals prevented *E. coli* EDL933 colonization. The results support a model in which invading pathogenic *E. coli* must compete with the gut microbiota to obtain the nutrients needed to colonize and establish infection; accordingly, the outcome of the challenge is determined by the aggregate capacity of the native microbiota to consume the nutrients required by the pathogen.

## Introduction

The gastrointestinal (GI) tract is home to a complex microbial community that has been implicated in both human health and disease. The GI tract is comparable to a chemostat in that the contents constantly turn over and to be successful in this highly competitive environment, an organism must reproduce at least as fast as the turnover rate or it will fail to colonize [1], [2]. Freter noted several factors that contribute to colonization success, and concluded that the most important is competition for resources, which led to the nutrient-niche hypothesis: in order to successfully colonize in the absence of adhesion to the mucosal surface an organism must use at least one limiting nutrient better than all other competitors [1], [3], [4], [5], [6].

Collectively, the human gut microbiota consists of 1,000 to 36,000 different species of bacteria [7], although the number in any one individual is thought to be between 500 and 1,000 species [8]. The gut microbiota is predominately comprised of anaerobic bacteria, mostly in the Firmicutes and Bacteriodetes phyla. *Escherichia coli* is the predominant facultative anaerobe in the mammalian GI tract, although other facultatives are present [9], [10]. Recent work from our laboratories suggests that *E. coli* exists in a symbiotic relationship with the anaerobic members of the gut microbiota. Through normal nutrient processing the anaerobes degrade complex polysaccharides, releasing the mono- and disaccharides *E. coli* needs for growth. In return, *E. coli* helps create an anaerobic environment by scavenging oxygen [11], [12]. This may explain why *E. coli* and other facultative anaerobes are the first colonizers of the infant gut [13], [14].

Once established, the gut microbiota is very stable, such that invading organisms often fail to colonize. This barrier effect is predicted by Freter's nutrient-niche hypothesis and is termed colonization resistance (CR) [10]. In a recent review, Stecher and Hardt explain that while the mechanisms of CR are incompletely understood, it is thought that they include direct inhibition of pathogens, the stimulation of host defenses, and nutrient depletion [15]. However, CR is not always effective, and invading species are sometimes able to compete with the microbiota, grow to sufficient numbers for colonization of the intestine, and cause disease. For example, *E. coli* pathogens infect humans, despite an average of 5 commensal strains of *E. coli* colonizing a human at any given time [16]. The disease burden of *E. coli* in the world is high and mostly carried by children in developing nations, where infantile diarrhea is a major factor in childhood mortality and up to 60% of cases can be attributed to two pathotypes of *E. coli* [17]. In developed nations, *E. coli* is not endemic, but disease outbreaks do occur, with approximately 5,000 cases of Shiga toxin-producing *E. coli* infection reported in the United States in 2011 [18]. In addition to enteric infections, disruptions in the gut microbiota have been indicated in inflammatory bowel disease [19], [20], and overgrowth of certain adherent and invasive *E. coli* has been associated with Crohn's disease [21], [22]. It is becoming increasingly apparent that a better understanding of the role of the commensal microbiota in CR is needed in order to effectively treat and prevent disease [23], [24].

Previously, we showed that *E. coli* MG1655, a commensal strain, and *E. coli* EDL933, an O157:H7 strain, use different sugars to colonize the streptomycin-treated mouse intestine [25]. Furthermore, we recently showed incomplete CR amongst different commensal *E. coli* strains to *E. coli* EDL933 invasion in the streptomycin-treated mouse model [26]. Although pre-colonization of individual commensal strains did not prevent colonization by *E. coli* EDL933, a combination of three commensal strains was effective [26]. On the basis of these findings, we hypothesized that a potential strategy for preventing colonization by enterohemorrhagic *E. coli* would be to pre-colonize mice with a combination of commensal strains that would fill the sugar-defined nutritional niches normally available to invading *E. coli* pathotypes. However, the sugar-defined niche of only one commensal strain has been characterized to date (16).

In order for such probiotic measures to be effective, the nutritional niches of both pathogenic and commensal strains must first be understood. The three commensal strains found to be the most effective at filling the nutritional niche of *E. coli* EDL933, in combination, were *E. coli* MG1655 (a K-12 human commensal strain), *E. coli* Nissle 1917 (a probiotic strain), and *E. coli* HS (a normal human commensal strain) [26]. *E. coli* Nissle 1917 was first isolated from an uninfected soldier during an outbreak of *Shigella* in 1917. The strain has been marketed for treatment of infectious diarrhea [27], and has shown to be as effective as current antibiotic treatments for the remission of ulcerative colitis [28] with no adverse effects reported [29]. *E. coli* Nissle 1917 lacks many of the virulence factors of pathogenic strains, but has several “fitness factors”, including anti-inflammatory properties, enhancement of host barrier function, and induction of the expression of human beta-defensins [30]. *E. coli* HS was first isolated in 1958 from a healthy human volunteer during a study of *Shigella flexneri* [31]. It is considered a true human commensal, colonizing humans strongly (10^10^ CFU/g feces) with no sign of disease [32]. Its genome recently was sequenced [33]. *E. coli* HS has been used extensively as a non-pathogenic control for human and animal studies of pathogenic *E. coli*, although little is known about the nature of *E. coli* HS *in vitro* or *in vivo*.

Despite its myriad benefits, the safety of *E. coli* Nissle 1917 as a probiotic has been questioned. Gronback *et al* showed that when both the host gut microbiota and adaptive immunity are defective in mice, *E. coli* Nissle 1917 was able to translocate through the epithelial layer, leading to dissemination, septicemia, and death of the animals [34]. The authors noted that although patients with incomplete gut microbiota have been the successful targets for probiotic treatment, cases have arisen where probiotics were associated with sepsis and other adverse effects, especially in immunocompromised patients [35], [36], [37], [38]. Also, when mice pre-colonized with only *E. coli* Nissle 1917 are fed a low dose of *E. coli* EDL933, the pathogenic strain is able to grow, albeit marginally, and persist in the intestine for the 11 day duration of the experiment [26]. Clearly, a greater understanding is needed of the physiology of commensal *E. coli* strains, keeping in mind that any positive effect against colonization of a pathogen may need to include multiple strains. In this study we determined the sugar-defined niches of *E. coli* strains HS and Nissle 1917, and compared them to those of the previously characterized strains, *E. coli* MG1655 and *E. coli* EDL933.

## Results

### Generation of sugar-negative strains

To determine which carbon sources are utilized for colonization by *E. coli* HS and Nissle 1917, mutants were constructed that were unable to utilize specific sugars for growth (Table 1). *E. coli* Nissle 1917 deletions were constructed via Lamda Red allelic replacement, as described previously [39]. Mutation frequency in *E. coli* HS by the routine Datsenko-Wanner method was low, as has been reported by others for non-K12 *E. coli* strains [40], [41]. We found an alternative Lambda Red plasmid, pKM208, which previously was shown to increase recombination efficiency in enterohemorrhagic strains of *E. coli* [40], to be superior for allelic replacement in *E. coli* HS.

**Table 1:** Bacterial strains and plasmids used in this study.

Strain or Plasmid	Genotype or phenotype^1^	Reference
HS	Wild type	[33]
HS Str^R^	Spontaneous Str^R^ mutant of HS	This Study
HS Str^R^Nal^R^	Spontaneous Nal^R^ mutant of HS Str^R^	This Study
Nissle 1917 Str^R^	Spontaneous Str^R^ mutant of Nissle 1917	[45]
EDL933 Str^R^Rif^R^	Spontaneous Rif^R^ mutant of EDL933 Str^R^	[46]
Strains derived from HS Str^R^:		
Δ*araBAD*	Δ*(araB-araD)::cat*	This Study
*araBAD+*	HS *araBAD*+ (restored to WT)	This Study
Δ*fucK*	Δ*fucK::kan*	This Study
Δ*galK*	Δ*galK::cat*	This Study
*galK+*	HS *galK*+ (restored to WT)	This Study
Δ*gntK ΔidnK*	Δ*gntK* Δ*idnK::cat*	This Study
Δ*lacZ*	Δ*lacZ::cat*	This Study
Δ*manA*	Δ*manA::cat*	This Study
Δ*nagE*	Δ*nagE::cat*	This Study
Δ*nanAT*	Δ*nanAT::cat*	This Study
Δ*rbsK*	Δ*rbsK::cat*	This Study
Δ*uxaC*	Δ*uxaC::kan*	This Study
Strains derived from Nissle 1917 Str^R^:		
Δ*agaWEFA*	Δ*(agaW- agaA)::cat*	This Study
Δ*araBAD*	Δ* (araB-araD)::cat*	This Study
Δ*fucK*	Δ*fucK::kan*	This Study
Δ*galK*	Δ*galK::cat*	This Study
Δ*gntK*	Δ*gntK::cat*	This Study
Δ*lacZ*	Δ*lacZ::cat*	This Study
Δ*manA*	Δ*manA::cat*	This Study
Δ*nagE*	Δ*nagE::cat*	This Study
Δ*nanAT*	Δ*nanAT::cat*	This Study
Δ*rbsK*	Δ*rbsK::cat*	This Study
Δ*uxaC*	Δ*uxaC::kan*	This Study
Plasmids:		
pKD46	T^s^ Amp^r^ P_araC_-λ-red recombinase	[39]
pKM208	T^s^ Amp^r^ *lacI* P_tac_-*red-gam-lacI*	[40]
pCP20	T^s^ Amp^r^ FLP recombinase	[39]

1 Str^R^, streptomycin resistance; Nal^R^, nalidixic acid resistance; Rif^R^, rifampicin resistance; Amp^R^, ampicillin resistance; T^S^, temperature sensitive replication.

### Confirmation of sugar-negative phenotypes

The goal of this study was to compare the in vivo (i.e., in the intestine) nutrition of *E. coli* commensal strains HS and Nissle 1917 with that of the pathogen *E. coli* EDL933. We previously assessed the ability of *E. coli* EDL933 to use 12 different sugars in the intestine [25]. We note that the hexuronates glucuronate and galacturonate are considered here to be one sugar because mutation of the hexuronate catabolism pathway eliminates growth on both sugars. Of these 12 sugars, neither *E. coli* HS nor Nissle 1917 have the genes for sucrose catabolism and *E. coli* HS also lacks the genes for N-acetylgalactosamine catabolism. Hence, mutations were constructed in the 11 sugar catabolism pathways possessed by *E. coli* Nissle 1917 and 10 pathways in *E. coli* HS.

**Figure 1:**
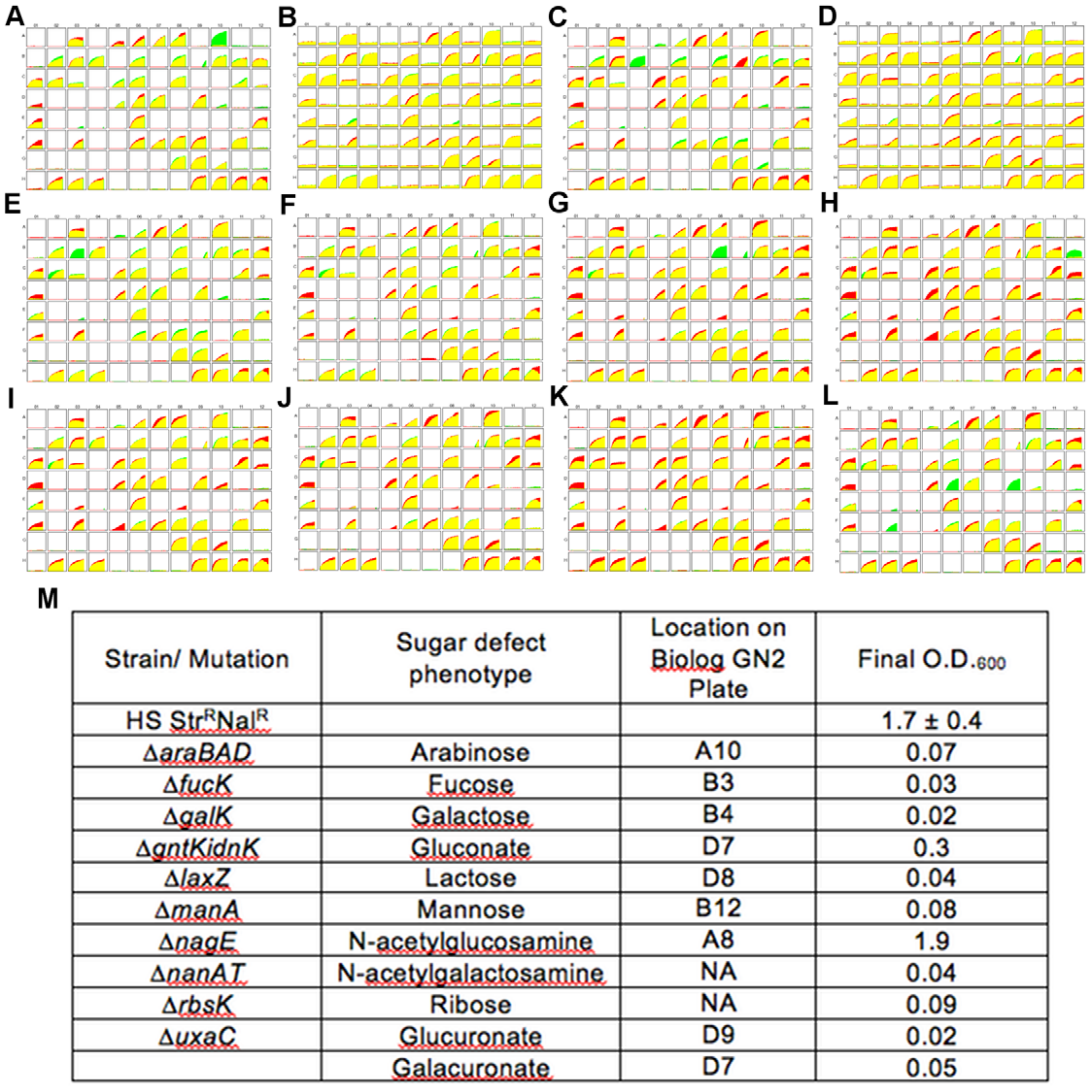
Phenotypic assay results for *E. coli* HS mutants. **A–L.** Biolog GN2 (Gram negative carbon nutrition) plates were utilized to assess the overall metabolic capacity of the mutants constructed in *E. coli* HS to consume 95 individual carbon sources (see Materials and Methods for details). The kinetic curves plot substrate oxidation vs. time. Green represents carbon source utilization by the *E. coli* HS wild type strain, red represents the mutant strain, and yellow represents utilization by both strains. Mutant strains of *E. coli* HS: (**A**) Δ*araBAD*, (**B**) *araBAD+*, (**C**) Δ*galK*, (**D**) *galK+*, (**E**) Δ*fucK*, (**F**) Δ*gntK* Δ*idnK*, (**G**) Δ*lacZ*, (**H**) Δ*manA*, (**I**) Δ*nagE*, (**J**) Δ*nanAT*, (**K**) Δ*rbsK*, and (**L**) Δ*uxaC*. **M.** Sugar utilization was assessed in minimal media containing 0.2% carbon source. The final O.D._600_'s following 12 hour incubations are shown. Note: the generation time of *E. coli HS* Δ*nagE* on N-acetylglucosamine is 2.6 h compared to 1 h for the wild type (see text for details). The sugars N-acetylneuraminate and ribose are not represented on the Biolog plate (NA).

All mutant genotypes were sequenced and the phenotypes confirmed by growth on MOPS minimal media and Biolog assays. Results of the phenotypic assays for the *E. coli* HS mutants are shown in Figure 1. Only one of the *E. coli* HS mutants showed significant growth on the sugar targeted for mutation, *E. coli* HS Δ*nagE*, which lacks the N-acetylglucosamine phosphotransferase system [42]. This gene was chosen for mutation instead of *nagA*, as strains lacking *nagA* have been shown to accumulate N-acetylglucosamine-6-phosphate, which is toxic to *E. coli* [9]. Strains lacking *nagE* are still able to transport N-acetylglucosamine via the mannose phosphotransferase system, ManXYZ, which has a broad substrate specificity that includes N-acetylglucosamine [42]. *nagE* deficient strains can also access N-acetylglucosamine through peptidoglycan recycling, although at lower levels [43]. Thus, deletion of *nagE* does not prevent growth on N-acetylglucosamine as the sole carbon and energy source, but the growth rate is significantly lowered. Comparison of growth curves on MOPS minimal medium with 0.2% N-acetylglucosamine showed that *E. coli* HS Δ*nagE* had a doubling time of 2.6 h, compared to 1 h doubling time for the wild type. We also found that *E. coli* HS Δ*gntK* Δ*idnK* retained the ability to grow, albeit poorly, on gluconate. With gluconate as the sole carbon and energy source *E. coli* HS Δ*gntK* Δ*idnK* reached a final O.D._600_ of 0.3 (Figure 1), compared to a final O.D._600_ of approximately 1.0 for wild type *E. coli* HS (data not shown). *E. coli* HS apparently possesses an alternative route for gluconate phosphorylation, despite deletion of the two known gluconate kinase genes [44].

Biolog assays confirmed the sugar-negative phenotypes of the remaining *E. coli* HS strains, as shown in Figure 1. Although not shown, the phenotypes of the *E. coli* Nissle 1917 mutants were identical to those of the *E. coli* HS mutants with the exception of *E. coli* Nissle 1917 Δ*gntK*, which was unable to grow on gluconate. For each sugar-negative mutant, Biolog assays indicated the use of other carbon sources was unaffected (Figure 1).

### *E coli* HS Str^R^ and HS Str^R^ Nal^R^ and *E. coli* Nissle 1917 Str^R^ and Nissle 1917 Str^R^ Nal^R^ are isogenic

To ensure that spontaneous mutations conferring nalidixic acid resistance do not cause inherent growth defects, equal numbers of *E. coli* HS Str^R^ and Str^R^ Nal^R^ were competed in the streptomycin-treated mouse model, as were *E. coli* Nissle 1917 Str^R^ and Str^R^ Nal^R^ [45], as described previously for *E. coli* EDL933 [46]. The *E. coli* HS Str^R^ and Str^R^ Nal^R^ strains co-colonized for 15 days at approximately 10^8^ CFU/g feces (data not shown) and thus are considered isogenic and can be regarded as wild type strains.

### The in vivo carbon nutrition of *E. coli* HS

**Figure 2:**
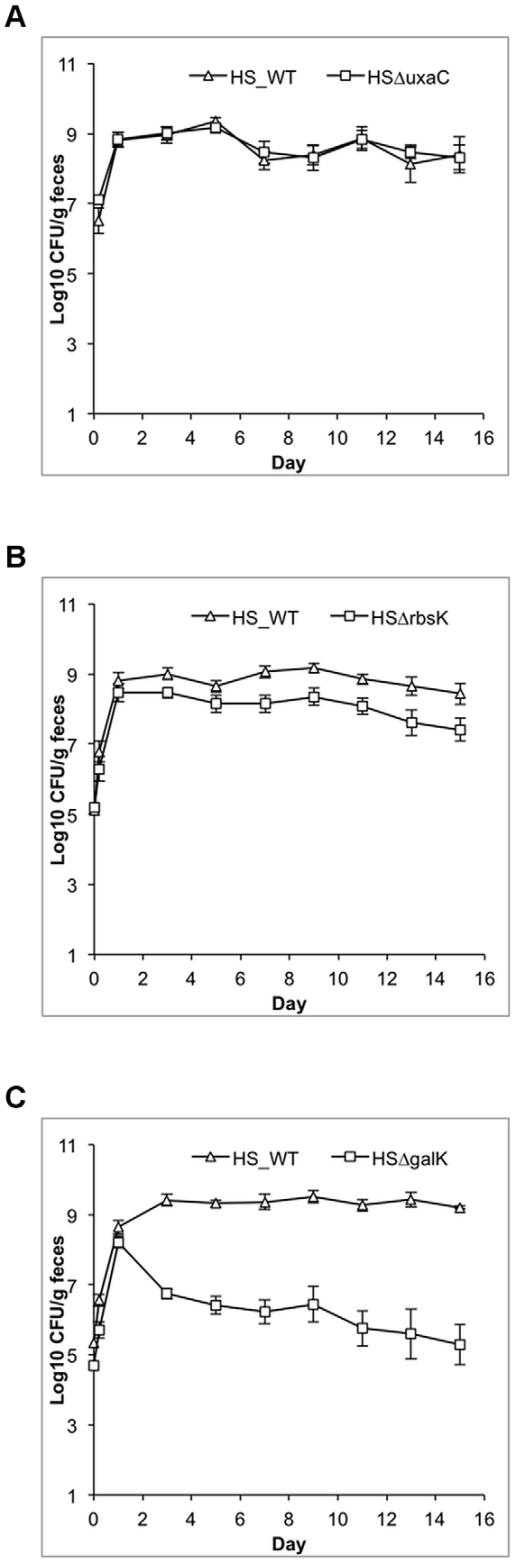
Competitive colonization of *E. coli* HS mutants vs. wild type. Sets of three mice were fed 10^5^ CFU of both the *E. coli* HS wild type strain and mutant strain. At the times indicated, fecal samples were homogenized, diluted, and plated as described in Materials and Methods. Compiled data from at least two distinct experiments (6 mice) are shown. Standard errors of the log_10_ means of CFU/gram feces are indicated with error bars. (**A**) Δ*uxaC*, which does not have a colonization defect; (**B**) Δ*rbsK*, which has a moderate colonization defect; (**C**) Δ*galK*, which has a severe colonization defect.

In order to test which carbon sources are important for intestinal colonization of *E. coli* HS, sugar-negative mutants were competed against their wild type parent strain in streptomycin-treated mice by feeding 10^5^ CFU of both strains. Results for the initiation phase (Day 1) and maintenance phase (Day 9), which best reflects the outcome of competition following initiation of colonization [9], are shown in Table 2. Representative plots of mutations causing no colonization defect, or causing moderate or severe defects are shown in Figure 2. For 6 of the 10 sugars tested, the mutation was shown to cause a statistically significant defect of the mutants compared to the wild type during the maintenance stage of colonization. The relative severity of the colonization defects of the *E. coli* HS mutants was galactose>arabinose>gluconate>N-acetylglucosamine>lactose>ribose. N-acetylneuraminate, fucose, mannose, and hexuronates were found to be unnecessary for growth of *E. coli* HS in the intestine.

**Table 2:** *E. coli* HS sugar utilization in the mouse intestine.

	Log10 difference on:		
Mutation	Day 1	Day 9	p Value
Δ*araBAD*	0.8±0.4	2.5±0.4	**2.61×10^−4^**
*araBAD*+	0.0±0.6	0.7±0.6	5.5×10^−1^
Δ*fucK*	0.4±0.3	0.0±0.4	9.1×10^−1^
Δ*galK*	0.4±0.4	3.1±0.7	**1.13×10^−3^**
*galK+*	0.5±0.1	0.4±0.1	3.5×10^−2^
Δ*gntK* Δ*idnK*	1.1±0.4	2.4±0.4	**2.06×10^−5^**
Δ*lacZ*	0.4±0.7	1.0±0.5	**4.56×10^−2^**
Δ*manA*	0.6±0.2	0.3±0.3	7.05×10^−1^
Δ*nagE*	0.4±0.4	1.9±0.4	**8.42×10^−5^**
Δ*nanAT*	0.8±0.5	1.3±0.7	7.62×10^−2^
Δ*rbsK*	0.3±0.5	0.8±0.4	**1.78×10^−2^**
Δ*uxaC*	0.0±0.4	0.1±0.6	8.45×10^−1^

The difference between population density of the wild type and each mutant strain is shown at Day 1 and Day 9, plus or minus the standard error of the mean. Significant values (bold) indicate log difference >0.8 and student's *t* test value P<0.05 at Day 9.

### Colonization defects are due to deletion of the targeted gene

To ensure that inability to co-colonize with the wild type parent was in fact due to the genetic disruption of the specific sugar utilization pathway and not a deleterious mutation at an unintended second site, complemented strains were constructed. To save mice, only two mutations were complemented. Previously, we found that complementation restored the wild type phenotype and eliminated the colonization defect, confirming that the defect was caused by the mutation and not a second site mutation [47]. In this study, *E. coli* HS Δ*araBAD* (arabinose negative) and *E. coli* HS Δ*galK* (galactose negative) were chosen for restoration of gene function because they showed the greatest defects when colonized against the wild type strain, a 2.5 and 3.1 log difference, respectively (Table 2). When these functions were restored by reintroduction of the wild type alleles, the complimented strains showed restoration of sugar utilization in Biolog assay (Figure 1), and no colonization defect in competition with the wild type (Table 2).

### The in vivo carbon nutrition of *E. coli* Nissle 1917

**Figure 3:**
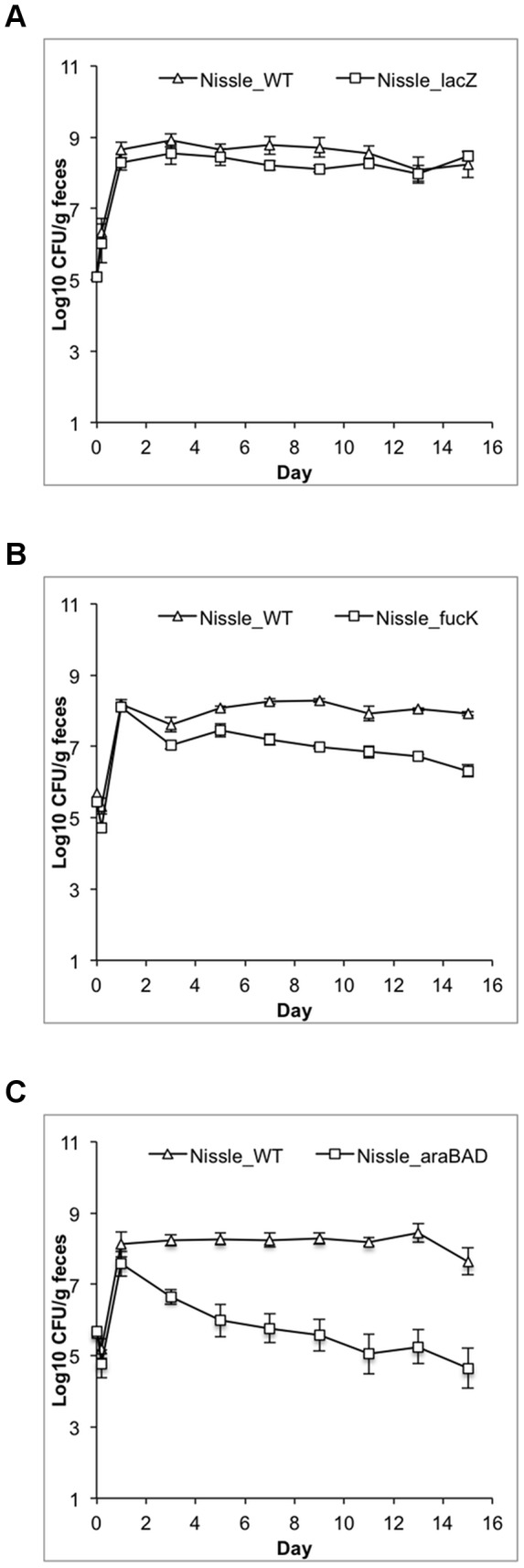
Competitive colonization of *E. coli* Nissle 1917 mutants vs. wild type. Data were collected as described in Figure 2. (**A**) Δ*lacZ*, which does not have a colonization defect; (**B**) Δ*fucK*, which has a moderate colonization defect; (**C**) Δ*araBAD*, which has a severe colonization defect.

Analogous to the experiments described above for *E. coli* HS, a series of metabolism mutants was competed against the wild type *E. coli* Nissle 1917 parent strain (Str^R^ Nal^R^). The results for initiation (Day 1) and maintenance (Day 9) phases in competitive colonizations are shown in Table 3. Representative plots of three colonization experiments are shown in Figure 3. For 7 of the 11 sugars tested, mutation was shown to cause a statistically significant defect in the maintenance stage of colonization. The relative severity of the colonization defects of the *E. coli* Nissle 1917 strains was arabinose>fucose>galactose>gluconate>N-acetylgalactosamine>mannose = N-acetylneuraminate. Of the sugars tested, only lactose, N-acetylglucosamine, the hexuronates, and ribose were found to be unnecessary for growth of *E. coli* Nissle 1917 in the intestine.

**Table 3:** *E. coli* Nissle 1917 sugar utilization in the mouse intestine.

	Log10 difference on:		
Mutation	Day 1	Day 9	p Value
Δ*agaWEFA*	0.1±0.1	1.0±0.2	**9.64×10^−3^**
Δ*araBAD*	0.5±0.4	2.7±0.3	**1.01×10^−3^**
Δ*fucK*	0.1±0.1	1.3±0.1	**2.33×10^−9^**
Δ*galK*	1.0±0.2	1.2±0.2	**2.74×10^−3^**
Δ*gntK*	0.6±0.2	1.1±0.2	**4.28×10^−3^**
Δ*lacZ*	0.4±0.2	0.6±0.2	7.85×10^−2^
Δ*manA*	0.4±0.2	0.9±0.3	**3.97×10−** 2
Δ*nagE*	0.0±0.3	0.0±0.2	8.77×10^1^
Δ*nanAT*	0.3±0.1	0.9±0.1	**3.45×10^−3^**
Δ*rbsK*	−0.1±0.1	0.2±0.1	1.06×10^−1^
Δ*uxaC* 2	−0.9±02	−0.6±0.1	4.92×10^−2^

The difference between population density of the wild type and each mutant strain is shown at Day 1 and Day 9, plus or minus the standard error of the mean. Significant values (bold) indicate log difference >0.8 and student's *t* test value P<0.05 at Day 9.

2 Significance refers to Nissle 1917Δ*uxaC* ability to outcompete wildtype.

### *E. coli* strains HS, Nissle 1917, MG1655, and EDL933 display different nutritional profiles in streptomycin-treated mice

Previously we demonstrated that *E. coli* MG1655 and *E. coli* EDL933 display different nutritional profiles in the streptomycin-treated mouse intestine (16). Table 4 compares the *in vivo* carbon preferences of four *E. coli* strains: *E. coli* MG1655, the K12 strain; the enterohemorrhagic O157:H7 type strain *E. coli* EDL933; *E. coli* Nissle 1917; and *E. coli* HS. Of the 12 sugars tested, *E. coli* EDL933 uses seven of them, in order of preference, ribose>galactose>sucrose>mannose>N-acetylglucosamine>arabinose>hexuronates (16). Therefore, *E. coli* EDL933 is able to use 2 sugars that are not used by the commensals: sucrose, which cannot be catabolized by the commensals because they lack the pathway, and the hexuronates, the loss of which has the least impact on colonization by *E. coli* EDL933. The results in Table 4 show that each strain displays a unique nutritional program in the streptomycin-treated mouse intestine.

**Table 4:** Sugar utilization in the intestine by *E. coli* strains.

Sugar-negative phenotype	Mutation	MG1655^3^	EDL 933^3^	Nissle 1917^4^	HS^4^
Arabinose	Δ*araBAD*	Yes	Yes	Yes	Yes
Fucose	Δ*fucK*	No	No	Yes	No
Galactose	Δ*galK*	No	Yes	Yes	Yes
Gluconate	Δ*gntK* Δ*idnK*	Yes	No	Yes	Yes
Hexuronates	Δ*uxaC*	No	Yes	No	No
Lactose	Δ*lacZ*	No	No	No	Yes
Mannose	Δ*manA*	No	Yes	Yes	No
N-acetylglucosamine	Δ*nagE*	Yes	Yes	No	Yes
N-acetylgalactosamine	Δ*agaWEFA*	NA^5^	No	Yes	NA
N-acetylneuraminate	Δ*nanAT*	Yes	No	Yes	No
Ribose	Δ*rbsK*	No	Yes	No	Yes
Sucrose	Δ*sacH*	NA	Yes	NA	NA

Results show the difference in population sizes of wild type verses mutant strains at Day 9. Yes indicates >0.8 log_10_ colonization difference and P<0.05.

3 Data from [25]. Fabich AJ, Jones SA, Chowdhury FZ, Cernosek A, Anderson A, et al. (2008) Comparison of carbon nutrition for pathogenic and commensal *Escherichia coli* strains in the mouse intestine. Infection and immunity 76: 1143–1152.

4 This study.

5 NA indicates that the pathway is not intact in this genetic background.

### *E. coli* HS and Nissle 1917 fill the sugar-defined niches occupied by EDL933

**Figure 4:**
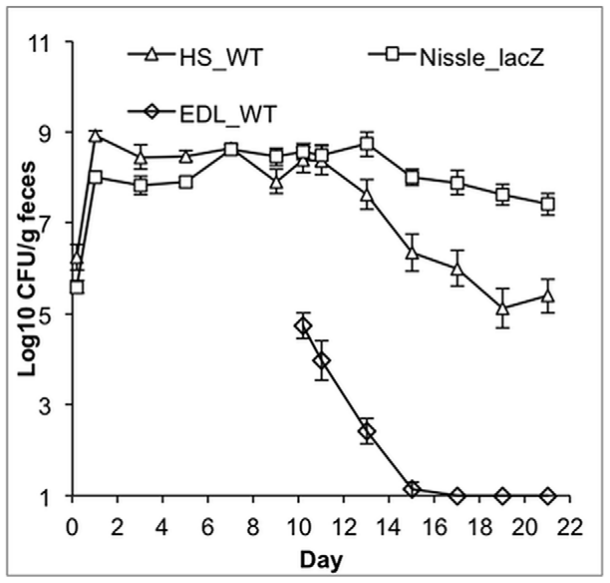
Mice precolonized with *E. coli* HS and *E. coli* Nissle 1917 prevent colonization by *E. coli* EDL 933. Mice were fed 10^5^ CFU of *E. coli* HS Str^R^Nal^R^ and *E. coli* Nissle 1917 Str^R^ Δ*lacZ::cat*. Ten days later, mice were fed 10^5^ CFU *E. coli* EDL933. Data were collected as described in Figure 2.

Previously we showed that pre-colonization with three commensal strains for 10 days was sufficient to prevent subsequent challenge by *E. coli* EDL933, which was completely eliminated from the intestine [26]. If the basis for exclusion of *E. coli* EDL933 in that experiment was because the commensals utilized the nutrients needed by *E. coli* EDL933 to compete and colonize, we reasoned that any commensal strain or combination of strains that effectively catabolizes the sugars used by *E. coli* EDL933 would prevent its colonization. Therefore, we tested the ability of *E. coli* EDL933 to colonize mice that were pre-colonized with *E. coli* HS and *E. coli* Nissle 1917, which the data in Table 4 indicated should be equally effective without MG1655 present. The two commensal strains were fed to mice at the beginning of the experiment, using nalidixic acid selection to enumerate *E. coli* HS Str^R^Nal^R^ and chloramphenicol selection to enumerate *E. coli* Nissle 1917 Δ*lacZ::cat* (as shown in Table 3 and Figure 3, the *lacZ* mutation does not affect its colonization). The precolonized animals were challenged on day 10 with *E. coli* EDL933 Str^R^Rif^R^ and the experiment was continued for 11 more days. The results of this experiment, shown in Figure 4, indicated that pre-colonization for 10 days with the two commensal *E. coli* strains did indeed completely prevent challenge by *E. coli* EDL933, which was eliminated 5 days following association. Thus, some but not all commensal *E. coli* strains exert CR against *E. coli* EDL933 in the mouse intestine.

## Discussion

The results from this study show that the human commensals *E. coli* HS and *E. coli* Nissle 1917 each occupy a unique nutritional niche in the mouse intestine (Table 4). Of the 12 sugars available in the mucus layer, *E. coli* HS has the genetic capacity to use 10 and actually utilizes 6 for colonization: galactose, arabinose, gluconate, N-acetylglucosamine, lactose, and ribose. Here, we also show that *E. coli* Nissle 1917 uses a differing list of 7 carbon sources to support colonization, including arabinose, fucose, galactose, gluconate, N-acetylglucosamine, and N-acetylneuraminate, and mannose. Previously we showed that *E. coli* MG1655 utilizes 5 sugars, arabinose, fucose, gluconate, N-acetylglucosamine, and N-acetylneuraminate [25]. Each of these commensals is capable of colonizing mice that were pre-colonized with one of the others [26], and each strain is capable of utilizing at least one sugar not used by the others in vivo, which suggests that differences in their in vivo sugar preferences allows them to occupy distinct nutrient-defined niches in the intestine.

The intestinal niche occupied by pathogenic *E. coli* EDL933 is at least partially defined by utilization of arabinose, galactose, hexuronates, mannose, N-acetylglucosamine, ribose, and sucrose for colonization [25]. Together, *E. coli* HS and *E. coli* Nissle 1917 utilize all but two of these carbon sources. *E. coli* HS was found to be the only strain, besides *E. coli* EDL933, which uses N-acetylglucosamine for colonization, whereas *E. coli* Nissle 1917 was found to be the only strain, besides *E. coli* EDL933, to use mannose (Table 4). Two sugars utilized by the pathogenic *E. coli* EDL933 but not utilized by the commensal strains tested are sucrose and the hexuronates. Only *E. coli* EDL933 has the genes necessary for sucrose catabolism. It is possible that *E. coli* EDL933 uses these sugars in order to initiate colonization, although the energy yield may not be enough to enable it to persist [25]. Leatham, et al., showed that when mice pre-colonized with three commensal *E. coli* strains were fed *E. coli* EDL933, it dropped from a population of about 10^5^ CFU/g feces to approximately 10^2^ CFU/g feces by 48 hours, and down to <10 CFU/g feces 10 days post association [26]. However, individually none of these commensal strains could prevent colonization of EDL933 [26]. Since the in vivo substrate range of *E. coli* Nissle 1917 and HS cover the same sugars without MG1655 in the mix, we predicted that the two strains would prevent EDL933 and found indeed this was the case (Figure 4). This result supports the hypothesis that nutrient consumption by certain *E. coli* strains can limit nutrient availability to other *E. coli* strains, supporting the idea that CR can be attributed, at least in part, to competition for nutrients.

It was important in this study to establish that the observed colonization defects are due to the loss of gene function for sugar utilization, and not due to second site mutations. Our results showed that the two mutations that caused the largest colonization defects in *E. coli* HS prevented growth on arabinose and galactose, respectively. Genetic complementation of these mutations (wild type *araBAD* was recombined into *E. coli* HS Δ*araBAD* and wild type *galK* into *E. coli* HS Δ*galK*) restored both complemented strains to the wild type colonization phenotype (Table 2), proving that the mutant colonization defects were due solely to mutation of the specific sugar pathway.

It is interesting that *E. coli* HS is the only *E. coli* strain found thus far to utilize lactose for colonization. This strain has often been used as a control during human and animal studies of pathogenic *E. coli* and *Salmonella* strains due to its ability to consistently colonize the intestine in high numbers [32], [48], [49], [50]. While further studies are needed, perhaps utilization of lactose is the reason for such strong colonization shown by *E. coli* HS. The amount of lactose present in the human large intestine differs from person to person, based on their diet and the presence or absence of lactase, the enzyme that degrades lactose [51]. It is well known that humans with decreased lactase production suffer from gastrointestinal symptoms such as bloating, gas, and diarrhea when excess lactose passes from the small intestine and is fermented by members of the gut microbiota [51]. Human adults are unique among mammals in having the ability to produce lactase, which most mammals lose upon weaning [52]. The results of this study suggest that *E. coli* HS can compete with the native gut microbiota for lactose.

The use of streptomycin treatment to remove native *E. coli* from the mice is required to overcome CR to experimentally introduced *E. coli* [10]. It is not possible to conduct the experiments in conventional mice, which are resistant to colonization by *E. coli*, nor would the experiments be possible in humans, who are colonized on average by five different commensal *E. coli* strains [16]. However, with the native *E. coli* removed by streptomycin treatment, the system allows the determination of in vivo nutrient utilization by introduced *E. coli* strains. Despite alterations to the mouse microbiota caused by streptomycin treatment, there remains a large and diverse anaerobe population in the intestine [10], [47]. We contend that results of the experiments described here extend to a more complex native microbiota found in conventional animals, including humans, and that the aggregate capacity of the microbiota to consume nutrients needed by invading bacteria to colonize is a component of CR, which is central to the probiotic concept [53].

Probiotics are defined as “live microorganisms which, when administered in adequate amounts, confer a health benefit on the host” [53]. In recent years, interest in their use to promote human health has increased dramatically. For almost a century, *E. coli* Nissle 1917 has been used as a probiotic for the treatment of infectious diarrhea [27]. In addition to its ability to occupy a different nutritional niche from that of *E. coli* EDL933, *E. coli* Nissle 1917 also has several fitness factors [30]. Nissle 1917 is also effective in maintaining remission in ulcerative colitis patients as traditional treatments [28], [29], [54], possibly through the increased production of indole [55]. However, Leatham, et al., showed that in the streptomycin-treated mouse model, pre-colonization with *E. coli* Nissle 1917 alone was insufficient to keep *E. coli* EDL933 from initiating colonization and persisting, albeit in low numbers [26]. This level of colonization may be high enough for *E. coli* EDL933 to reach the epithelium and express virulence factors. There has been no work, to date, to indicate that *E. coli* HS benefits humans. However, the strain has no adverse effects when fed to humans at high doses (10^10^ CFU) and has been used extensively as a representative of a “normal” commensal strain in human studies, with no ill outcomes [32], [48], [49], [50]. The new finding reported here, that *E. coli* Nissle 1917 and HS prevent colonization by EDL933, supports the hypothesis that nutrient consumption by commensal *E. coli* can limit nutrient availability to pathogens, which in turn points to the potential of probiotics for preventing disease.

## Materials and Methods

### Bacterial strains and growth conditions

Bacterial strains and plasmids used in this study are listed in Table 1. *E. coli* HS was obtained from David Rasko at the University of Maryland [33]. Derivation of the *E. coli* Nissle 1917 parent strains used in this study was described previously [45]. The *E. coli* EDL933 parent strain used in this study also was described previously [46]. Cultures were grown on Luria-Bertani (LB) broth at 250 RPM gyratory shaking or on LB agar. All cultures were incubated at 37°C, except those containing temperature-sensitive replicon plasmids, which were grown at 30°C. Antibiotics were supplemented as required to the following final concentrations: streptomycin, 100 µg/ml, nalidixic acid, 50 µg/ml, chloramphenicol, 30 µg/ml, kanamycin, 40 µg/ml, amplicillin, 100 µg/ml, rifampicin, 50 µg/ml.

### Electroporation and gene replacement

Null alleles were constructed in *E. coli* Nissle 1917 by using the Lambda Red allelic replacement system, first described by Datsenko and Wanner [39]. In our hands, successful allelic replacement in *E. coli* HS by the Datsenko and Wanner approach was rare, so instead we used the approach of Murphy and Campellone [40], modified as follows. To increase recombination efficiency, null alleles previously constructed in *E. coli* MG1655 [25] were used as a template for creating deletions in *E. coli* HS, which was possible because their genomes are highly conserved. The null alleles containing antibiotic resistance cassettes were PCR amplified from the genome of the appropriate *E. coli* MG1655 mutant, together with 500 to 700 base pairs of upstream and downstream flanking sequences, which increases recombination efficiency [41]. The PCR amplicons were purified using Qiagen PCR purification kit and electroporated into competent *E. coli* HS cells containing the Lambda Red pKM208 plasmid, according to the Murphy protocol [40]. Cells were recovered in super optimal broth (SOB) medium at 37°C with shaking for two hours and then plated on LB agar containing appropriate antibiotics. In this way the target genes on the *E. coli* HS genome were deleted and replaced with chloramphenicol or kanamycin resistance cassettes, which acted as selectable markers in colonization experiments. Single gene deletions began with deletion of the start codon and ended with the stop codon. Deletions of contiguous genes within an operon began with the start codon of the first gene and ended with the stop codon of the last gene deleted. Strains with multiple mutations at different loci were created by sequential allelic replacement. The first gene was replaced with a resistance cassette, which was removed using FLP recombinase [39]. The second gene was then replaced with a resistance cassette, leaving the selectable marker in place of the second gene deleted. All constructions were verified by DNA sequencing and phenotypic analysis.

### Phenotypic analysis

For confirmation of phenotypes, putative mutant strains and their wild type parents were grown in 3-*N*-morpholino propanesulfonic acid (MOPS) defined minimal medium [56] with 0.2% carbon source at 37°C with gyratory shaking for 12–14 hours. Cell growth was determined spectrophotometrically at OD_600_. To demonstrate the slower growth phenotype of the *E. coli* HS Δ*nagE* mutant, the growth rates in MOPS minimal medium plus 0.2% N-acetylglucosamine were measured for the mutant and the wild type. In addition, Biolog assays were used to determine the metabolic capacity of each deletion strain in *E. coli* HS, as described previously [57]. Biolog GN2 (Gram negative carbon nutrition) plates were used according to manufacturer's instructions. In brief, strains were grown overnight on tryptic soy agar, suspended in Biolog inoculating fluid (gellan gum, 0.2 g/liter; NaCL, 4.0 g/liter; Pluronic F-68, 0.3 g/liter; 2.5 mM thioglycolate) to a final OD_600_ of 0.2 to 0.3. Each well of the Biolog GN2 plate was inoculated with 150 µl cell suspension and incubated at 37°C for 24 hours in an Omnilog system. Tetrazolium violet dye reduction was measured at 15 min intervals and plotted vs. time, resulting in a kinetic curve. The area under the curve served as a measure of oxidation of each carbon source.

### Mouse colonization experiments

The streptomycin-treated mouse model was used to investigate the *in vivo* carbon nutrition of *E. coli* HS and Nissle 1917. This strategy for the study of intestinal colonization has been used extensively by our group [9], [11], [12], [25], [26], [45], [46], [57], [58]. Briefly, streptomycin treated water (5 g/L) was given to three male, six week old, CD-1 mice for 24 hours, clearing the intestine of native facultative anaerobic bacteria [10]. Streptomycin is an aminoglycoside antibiotic that inhibits protein synthesis, affecting terminal respiration pathways, carbohydrate metabolism, and cell division [59], [60], [61]. Anaerobic bacteria apparently do not take up aminoglycosides, making them intrinsically resistant [62]. Streptomycin is taken up by actively respiring cells and therefore selectively removes facultative anaerobic bacteria in the gut [63], [64]. Thus, streptomycin treatment opens a niche for the colonization of experimentally introduced *E. coli* strains while leaving the general population of anaerobic bacteria essentially intact [10]. Recent work from our laboratory using 16S rRNA gene sequencing showed that, while streptomycin treatment alters the anaerobic gut microbiota, the resulting microbial community is highly diverse, more so than any other animal model currently available [47].

As described previously [9], [11], [12], [25], [26], [45], [46], [57], [58], seed cultures were grown in LB liquid medium for eighteen hours prior to association. Food and water were withheld from the mice for fourteen hours prior to association. Mice were placed into separate cages and given 1 ml of a bacterial suspension prepared in 20% sucrose containing approximately 10^5^ CFU of each strain. Mice took up the inoculum orally, after which food and streptomycin treated water were returned *ad libidum* for the remainder of the experiment. At 5 and 24 hours post association and every other day thereafter, 1 gram of feces was diluted in 10 ml of 1% tryptone, homogenized, serially diluted, and plated onto MacConkey agar supplemented with appropriate antibiotics (typically streptomycin and nalidixic acid for wild type strain and streptomycin and chloramphenicol or kanamycin for mutant strains). The limit of detection was 10^1^ CFU/g feces. For experiments in which the challenge strain was associated with the mice on day 10, food and water again are withheld for 14 h and then 10^5^ CFU of the challenge strain was fed as described above, at which time food and water were returned. When necessary to differentiate strains, 100 colonies were tooth-picked from MacConkey streptomycin plates to MacConkey streptomycin and nalidixic acid plates. Colonizations were replicated at least twice, and the average population sizes and standard error of the mean of six or more animals was calculated.

### Gene complementation

To prove that selected colonization defects were in fact due to the targeted gene and not inadvertent second site mutations, the mutations were complemented in *E. coli* HS. Lambda Red plasmid pKM208 was electroporated into the mutated strain of choice, and a gene fragment containing the deleted allele was electroporated into competent cells as described above. Complemented strains were rescued by plating on MOPS minimal agar containing 0.2% of the appropriate carbon source. Strains were then verified via phenotypic and Biolog analysis, as described above. Complimented strains (streptomycin resistant) were colonized in the streptomycin-treated mouse model as above against the wild type strain (streptomycin and nalidixic acid resistant). Fecal samples were plated onto MacConkey agar supplemented with streptomycin. To distinguish the complemented strains from the wild type, 100 colonies were tooth-picked from the streptomycin plate to MacConkey agar containing streptomycin and nalidixic acid.

### Ethics statement

Animal experiments were conducted in accordance with the United States Department of Health and Human Services Office of Laboratory Animal Welfare. We used the minimum number of animals needed to obtain statistically significant results. To our knowledge, there is no in vitro experiment design that provides a reasonable alternative to animal experiments. The mice were cared for in a humane manner according to local, state, and federal regulations. During colonization experiments, there was no discomfort to the mice. Throughout the duration of the colonization experiments the cages were changed daily and the mice were provided food and water ad libitum. Mice were killed by CO_2_ asphyxiation at the conclusion of colonization experiments, consistent with recommendations of the Panel on Euthanasia of the American Veterinary Medical Association. The University of Oklahoma IACUC Approval Number is A3240-01, approved 01/25/2008. The University of Rhode Island IACUC Approval Number is A3690-01, approved 03/19/2007.
